# Supplementary material for: Anoctamin-1 is a core component of a mechanosensory anion channel complex in C. elegans
Source: Nat Commun. 2025 Feb 16;16:1680. doi: 10.1038/s41467-025-56938-z (PMC11830769; doi:10.1038/s41467-025-56938-z)
Supplement: Supplementary file 1 — Supplementary Information [file 41467_2025_56938_MOESM1_ESM.pdf]

# Supplementary Materials for

## Anoctamin-1 is a core component of a mechanosensory anion channel complex in *C. elegans*

Wenjuan Zou et al.

*Corresponding author. Wenjuan Zou, Email: zouwenjuan2008@163.com

*Corresponding author. Lijun Kang, Email: kanglijun@zju.edu.cn

**The supplementary file includes:**

Supplementary Figures 1 to 8

# Supplementary figures and figure legends

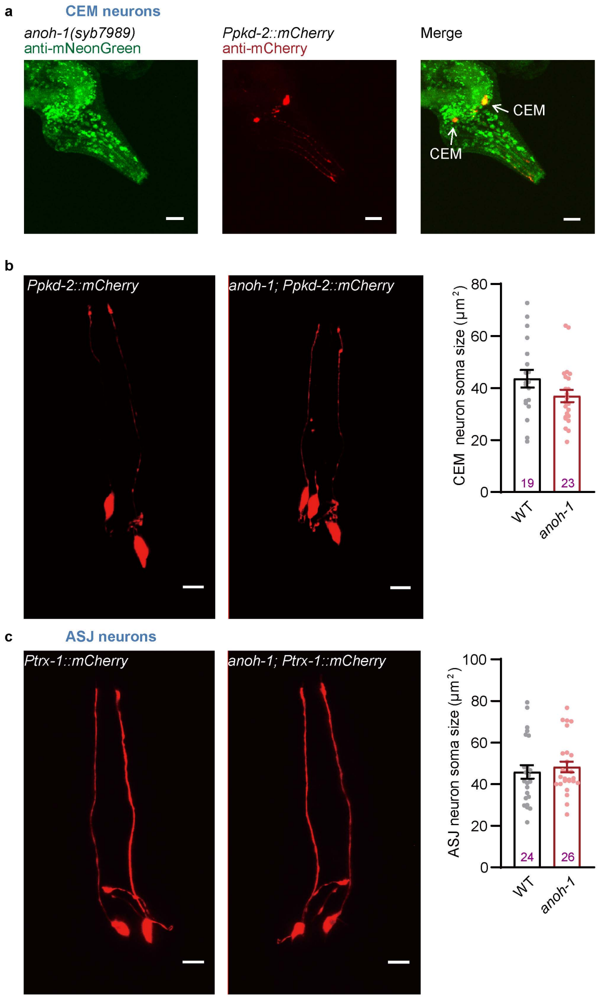


**Supplementary Fig. 1: The expression of ANOH-1 in CEM neurons, and its lack of effect on neuronal development.**

**a**, Anti-mNeonGreen and anti-mCherry staining in the worms with *anoh-1::mNeonGreen* knock-in and *Ppkd-2::mCherry* shows the expression of ANOH-1 in CEM neurons. Scale bars, 10 μm. **b**, Loss of ANOH-1 did not affect the development or cell shape of ASJ neurons. Left: morphologies of ASJ neurons; Right: size of ASJ soma. Scale bars, 10 μm. **c**, Loss of ANOH-1 did not affect the development or cell shape of CEM neurons. Left: morphologies of CEM neurons; Right: size of CEM soma. Scale bars, 10 μm. ASJ neurons were from Day 2 hermaphroditic animals, and CEM neurons were calculated from Day 2 adult male worms. Each dot represents 1 animal. Data are presented as mean ± SEM. **ns**: not significant. Source data are provided as a Source Data file.

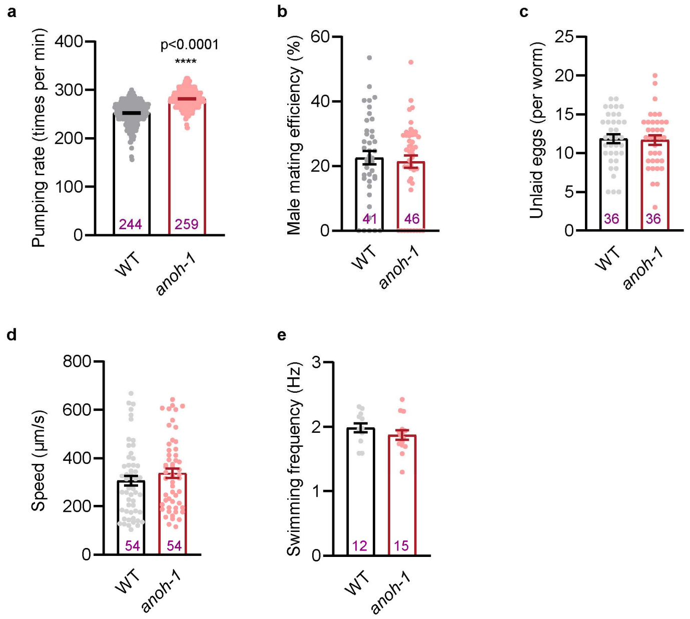


**Supplementary Fig. 2: ANOH-1 is involved in the regulation of pumping rate.**

**a**, The absence of ANOH-1 accelerated the pumping rate of adult Day 1 hermaphroditic animals. P values were calculated using the Mann-Whitney test. p<0.0001. **b**, Male mating efficiency was not changed in *anoh-1* mutant male worms. **c**, The number of unlaid eggs in adult Day 2 hermaphroditic animals was not changed in the *anoh-1* mutant hermaphroditic worms. **d**, The locomotion speed of adult Day 1 hermaphroditic animals was not changed in the *anoh-1* mutant background. **e**, The swimming frequency of adult Day 1 hermaphroditic animals in bath solution was not changed in the anoh-1 mutant background. Each dot represents 1 animal. Data are presented as mean ± SEM. ****P < 0.0001. Source data are provided as a Source Data file.

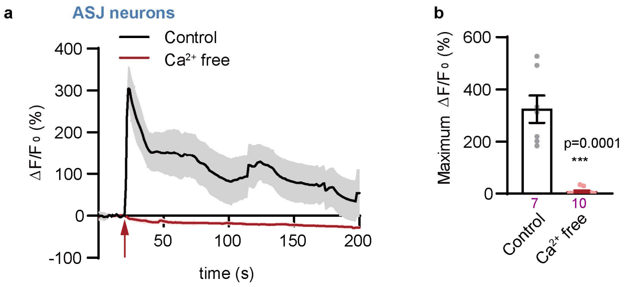


**Supplementary Fig. 3: Mechanical stimuli-evoked calcium responses in ASJ neurons are dependent on extracellular calcium.**

Mechanical stimuli-evoked calcium transients in ASJ neurons were abolished in a calcium-free bath solution. **a**: calcium responses; **b**: maximum $\Delta F/F_0$ changes. P values were calculated using the Mann-Whitney test. Day 2 adult hermaphroditic animals were used in these experiments. Each dot represents 1 animal. Data are presented as mean ± SEM, $^{***}P < 0.001$. Source data are provided as a Source Data file.

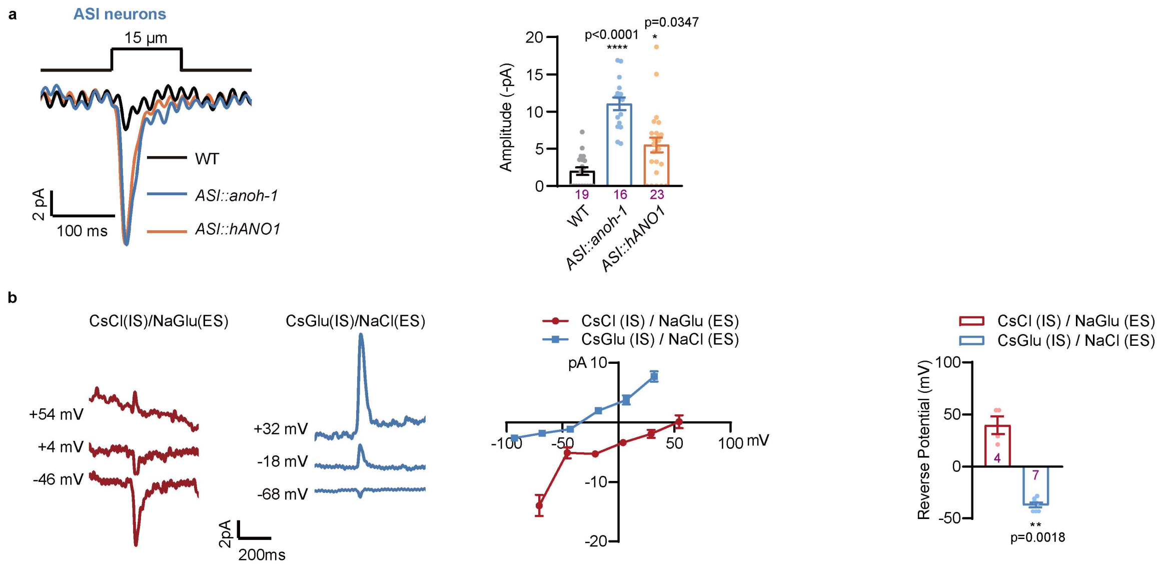


**Supplementary Fig. 4: Ectopic expression of nematode ANOH-1 or human ANO1 confers mechanosensitivity to ASI neurons.**

**a**, MRCs of ASI neurons ectopically expressing worm ANOH-1 or human ANO1 in response to nose touch. Left: sample traces; Right: peak MRC amplitudes. Mechanical stimulation: 15 µm displacement. Holding potential: -70 mV. P values were calculated using the Kruskal-Wallis test. **b**, MRCs recorded in ASI neurons ectopically expressing ANOH-1 are dependent on both intracellular (IS) and extracellular (ES) $Cl^-$ concentrations. Left: representative traces of MRCs. The cell membrane was initially voltage-clamped at +50 mV, 0 mV, and -50 mV, with the displayed voltages corrected posthoc for liquid junction potentials (LJPs). Middle: *I-V* relationship of MRCs. Left: the reversal potential of MRCs. Mechanical stimulation: 15 µm displacement. P values were calculated using the Welch's t test. Day 2 adult hermaphroditic animals were used in these experiments. Each dot represents 1 animal. Data are presented as mean ± SEM. *P < 0.05, **P < 0.01, ****P < 0.0001. Source data are provided as a Source Data file.

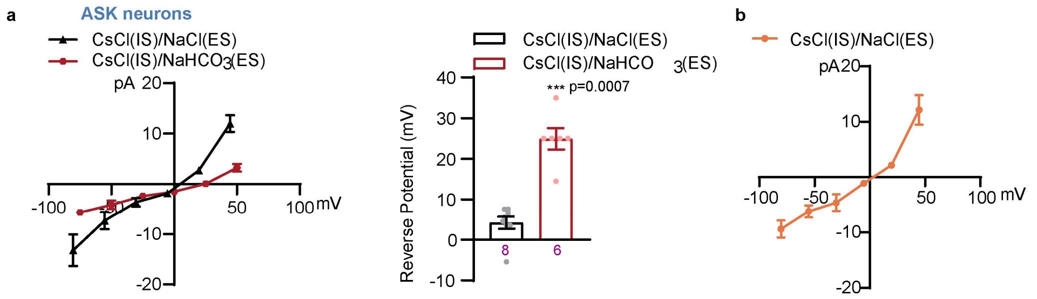


**Supplementary Fig. 5: I-V relationship of MRCs in ASK neurons ectopically expressing ANOH-1/ANO1.**

**a**, I-V relationship of MRCs in ASK neurons ectopically expressing ANOH-1, recorded with different intra- or extracellular solutions. Mechanical stimulation: 15 μm displacement. P values were calculated using the Mann-Whitney test. **b**, I-V relationship of MRCs in ASK neurons ectopically expressing human ANO1. Each dot represents 1 animal. Data are presented as mean ± SEM. ***P < 0.001. Source data are provided as a Source Data file.

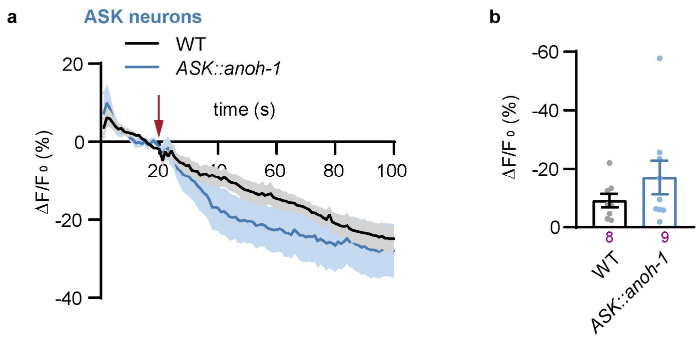


**Supplementary Fig. 6: Mechanical stimulation did not evoke calcium increases in ASK neurons with ectopic expression of ANOH-1.**

**a**, Calcium responses; **b**, Maximum $\Delta F/F_0$ changes. The arrow indicates the time of mechanical stimulation (15 μm displacement). Day 2 adult hermaphroditic animals were used in these experiments. Each dot represents 1 animal. Data are presented as mean ± SEM. Source data are provided as a Source Data file.

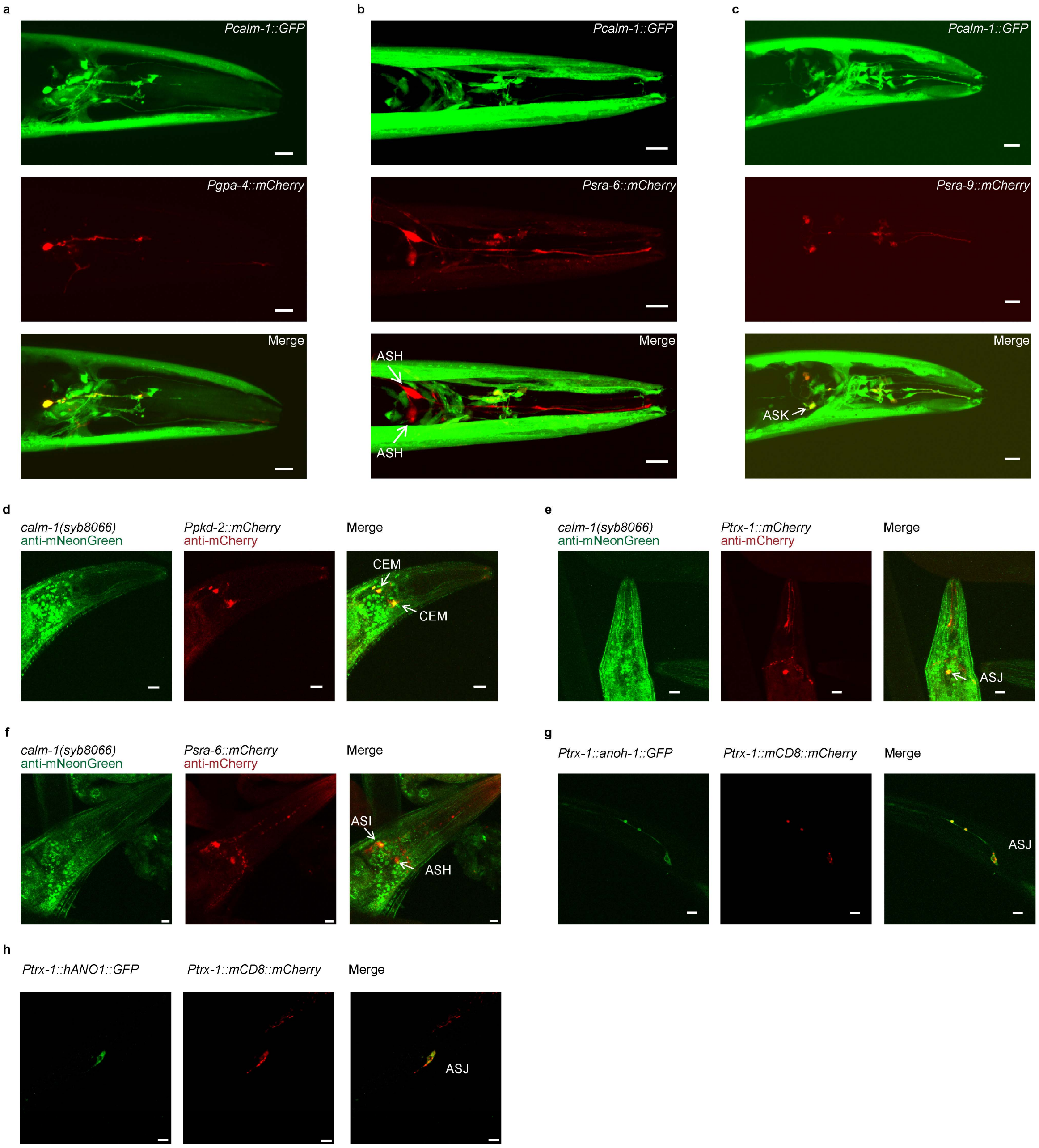


**Supplementary Fig. 7: The expression of CALM-1 in ASI, ASK, CEM, and ASJ neurons.** **a-c**, The co-localization of *Pcalm-1::GFP* with *Pgpa-4::mCherry* (a) and *Psra-9::mCherry* (c) suggests the expression of CALM-1 in ASI and ASK neurons. *Pcalm-1::GFP* is not detectable in ASH neurons. Scale bar: 10 μm. **d-f**, anti-mNeonGreen and anti-mCherry staining in worms strains with *calm-1::mNeonGreen* knock-in and *Ppkd-2::mCherry, Ptrx-1::mCherry*, or *Psra-6::mCherry* suggest the expression of CALM-1 in CEM, ASJ neurons, but not in ASH neurons. Scale bar: 10 μm.

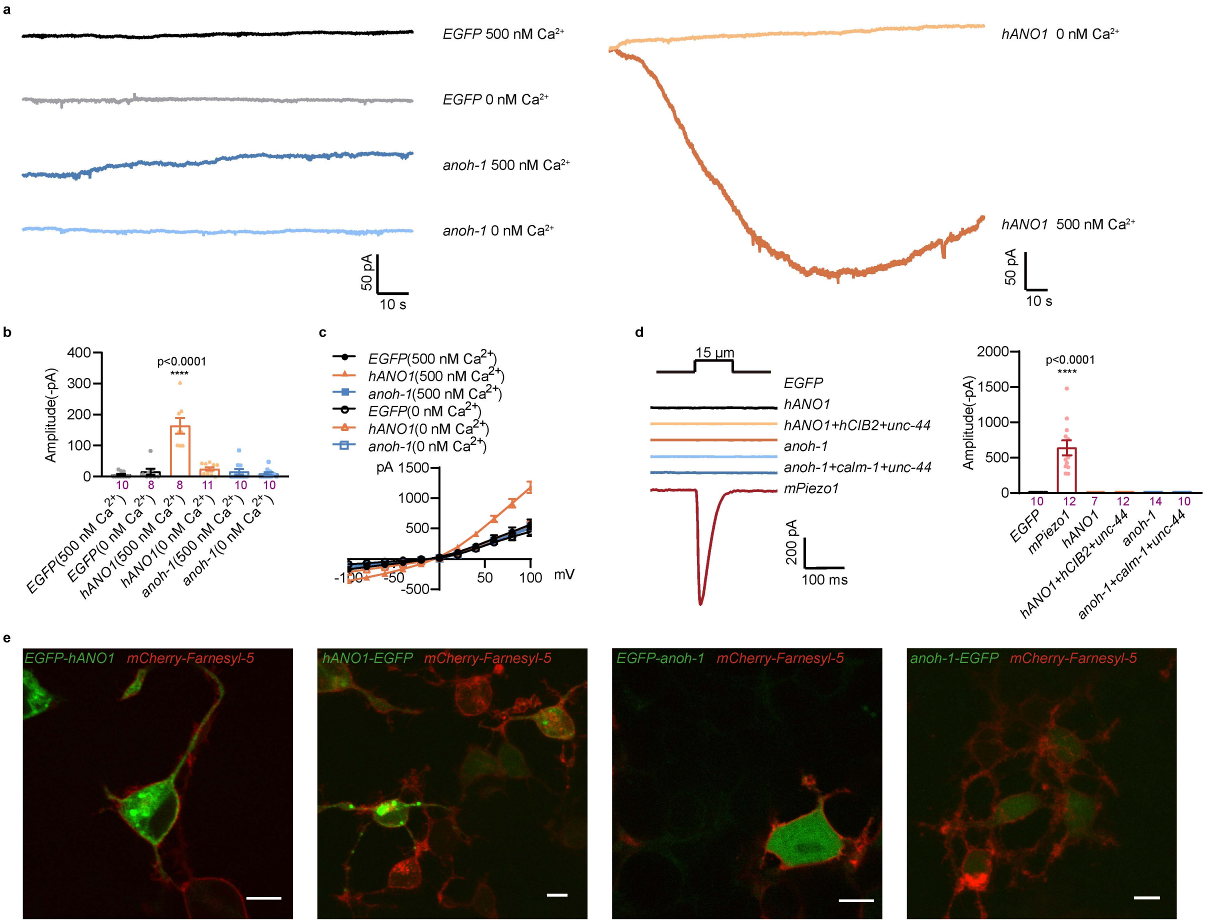


**Supplementary Fig. 8: Recordings in HEK-P1KO cells transfected with ANOH-1/ANO1**

**a- c**, $Ca^{2+}$-activated currents were recorded in HEK-P1KO cells cell transfected with human ANO1, but not with nematode ANOH-1. The patch pipette contained either 0 or 500 nM calcium. a, representative traces. b, peak amplitudes. c, the I-V relationship, indicating that the ANO1-dependent $Ca^{2+}$-activated currents are outwardly rectifying. P values were calculated using Kruskal-Wallis test. **d**, MRCs recordings in HEK-P1KO cells transfected with different plasmids. a, representative traces. b, peak amplitudes. P values were calculated using Kruskal-Wallis test. **e**, localization of N- or C-terminal EGFP-tagged human ANO1 and nematode ANOH-1 transfected into HEK-P1KO cells, co-labeled with the membrane marker mCherry-Farnesyl-5. Scale bar: 10 μm. **f**, localization of overexpressed nematode ANOH-1 and hANO1, and the membrane marker mCD8 in ASJ neurons. Scale bar: 10 μm. Each dot represents 1 single cell. Data are presented as mean ± SEM. ****$P < 0.0001$. Source data are provided as a Source Data file.
