# Supplementary material for: Anoctamin-1 is a core component of a mechanosensory anion channel complex in C. elegans
Source: Nat Commun. 2025 Feb 16;16:1680. doi: 10.1038/s41467-025-56938-z (PMC11830769; doi:10.1038/s41467-025-56938-z)
Supplement: Supplementary file 2 — Reporting Summary [file 41467_2025_56938_MOESM2_ESM.pdf]

nature portfolio

Corresponding author(s): Lijun Kang

Last updated by author(s): Jan 29, 2025

Reporting Summary

Nature Portfolio wishes to improve the reproducibility of the work that we publish. This form provides structure for consistency and transparency in reporting. For further information on Nature Portfolio policies, see our Editorial Policies and the Editorial Policy Checklist.

Statistics

For all statistical analyses, confirm that the following items are present in the figure legend, table legend, main text, or Methods section.

n/a	Confirmed	
☐	☒	The exact sample size (*n*) for each experimental group/condition, given as a discrete number and unit of measurement
☐	☒	A statement on whether measurements were taken from distinct samples or whether the same sample was measured repeatedly
☐	☒	The statistical test(s) used AND whether they are one- or two-sided<br>*Only common tests should be described solely by name; describe more complex techniques in the Methods section.*
☒	☐	A description of all covariates tested
☐	☒	A description of any assumptions or corrections, such as tests of normality and adjustment for multiple comparisons
☐	☒	A full description of the statistical parameters including central tendency (e.g. means) or other basic estimates (e.g. regression coefficient) AND variation (e.g. standard deviation) or associated estimates of uncertainty (e.g. confidence intervals)
☐	☒	For null hypothesis testing, the test statistic (e.g. *F*, *t*, *r*) with confidence intervals, effect sizes, degrees of freedom and *P* value noted<br>*Give P values as exact values whenever suitable.*
☒	☐	For Bayesian analysis, information on the choice of priors and Markov chain Monte Carlo settings
☒	☐	For hierarchical and complex designs, identification of the appropriate level for tests and full reporting of outcomes
☒	☐	Estimates of effect sizes (e.g. Cohen's *d*, Pearson's *r*), indicating how they were calculated

Our web collection on statistics for biologists contains articles on many of the points above.

Software and code

Policy information about availability of computer code

Data collection	Olympus IX71 microscope; Andor DL-604M EMCCD camera; Olympus FV1000 confocal laser microscope; Micro-Manager software; HEKA EPC-10 Amplifier
Data analysis	Image J software; Olympus FV10-ASW Viewer; Patch Master

For manuscripts utilizing custom algorithms or software that are central to the research but not yet described in published literature, software must be made available to editors and reviewers. We strongly encourage code deposition in a community repository (e.g. GitHub). See the Nature Portfolio guidelines for submitting code & software for further information.

Data

Policy information about availability of data

All manuscripts must include a data availability statement. This statement should provide the following information, where applicable:

- Accession codes, unique identifiers, or web links for publicly available datasets
- A description of any restrictions on data availability
- For clinical datasets or third party data, please ensure that the statement adheres to our policy

Source data of the Figures and Supplementary Figures are provided with this paper. All other data supporting the findings of this study are available from the authors on request.

Research involving human participants, their data, or biological material

Policy information about studies with human participants or human data. See also policy information about sex, gender (identity/presentation), and sexual orientation and race, ethnicity and racism.

Reporting on sex and gender	NA
Reporting on race, ethnicity, or other socially relevant groupings	NA
Population characteristics	NA
Recruitment	NA
Ethics oversight	NA

Note that full information on the approval of the study protocol must also be provided in the manuscript.

Field-specific reporting

Please select the one below that is the best fit for your research. If you are not sure, read the appropriate sections before making your selection.

☒ Life sciences ☐ Behavioural & social sciences ☐ Ecological, evolutionary & environmental sciences

For a reference copy of the document with all sections, see nature.com/documents/nr-reporting-summary-flat.pdf

Life sciences study design

All studies must disclose on these points even when the disclosure is negative.

Sample size	No statistical method was used to determine sample size. We determined sample size based on our previous experience with the experimental model and techniques, which are consistent with typical sample sizes used in the field. For each experiment, the exact n number is provided in the legends and bar graphs.
Data exclusions	No data were excluded from any of the experimental studies presented in the manuscript.
Replication	All experiments in this study were biologically repeated at least three times independently and all attempts at replication were successful.
Randomization	Caenorhabditis elegans were grouped according to genotype and were randomly chosen from siblings for all studies
Blinding	The data collection and analysis were blinded to group allocation.

Reporting for specific materials, systems and methods

We require information from authors about some types of materials, experimental systems and methods used in many studies. Here, indicate whether each material, system or method listed is relevant to your study. If you are not sure if a list item applies to your research, read the appropriate section before selecting a response.

Materials & experimental systems

n/a	Involved in the study	
☐	☒	Antibodies
☐	☒	Eukaryotic cell lines
☒	☐	Palaeontology and archaeology
☐	☒	Animals and other organisms
☒	☐	Clinical data
☒	☐	Dual use research of concern
☒	☐	Plants

Methods

n/a	Involved in the study	
☒	☐	ChIP-seq
☒	☐	Flow cytometry
☒	☐	MRI-based neuroimaging

Antibodies

Antibodies used	Anti-β-Tubulin (M1305-2; Huaan Hangzhou, 1:5,000), Anti-HA nanobody agarose beads: (KTSM1305, AlpaLifeBio), Anti-Flag beads: (KTSM1308, AlpaLifeBio), Anti-HA-Tag-HRP-direct (M180-7, MBL, 1:5,000), Anti-Flag-Tag-HRP-direct (M185-7, MBL, 1:5,000) were used in Co-IP, and anti-mNeuoGreen (A24858, Abclonal, 1:100), anti-mCherry (E022110-03, EarthOx, 1:500), Abclonal Alexa 488 (AS053, Abclonal, 1:300) and Alexa 594 (A11005, Thermo Fisher Scientific, 1:1,000) were used in immunostaining.

Validation	All antibodies were purchased from commercial vendors, who provided validation information on their website.

Eukaryotic cell lines

Policy information about cell lines and Sex and Gender in Research

Cell line source(s)	HEK293T cells (ATCC CRL-3216) and HEK-P1KO cells (ATCC CRL-3519) were cultivated with standard protocols
Authentication	The cell lines were not authenticated.
Mycoplasma contamination	The cell lines were free of mycoplasma contamination.
Commonly misidentified lines (See ICLAC register)	No commonly misidentified cell lines were used.

Animals and other research organisms

Policy information about studies involving animals; ARRIVE guidelines recommended for reporting animal research, and Sex and Gender in Research

Laboratory animals	Caenorhabditis elegans (all worms used in this paper are adult)
Wild animals	No wild animal was used in this study
Reporting on sex	Hermaphroditic and male worms were used in this study, and the sex used in each experiment was provided in the figure legends.
Field-collected samples	This study does not include samples from the field. All C. elegans strains were listed in the Methods, and were maintained and used according to standard protocols.
Ethics oversight	NA

Note that full information on the approval of the study protocol must also be provided in the manuscript.

Plants

Seed stocks	NA
Novel plant genotypes	NA
Authentication	NA
